# Swainsonine, an alpha-mannosidase inhibitor, may worsen cervical cancer progression through the increase in myeloid derived suppressor cells population

**DOI:** 10.1371/journal.pone.0213184

**Published:** 2019-03-06

**Authors:** Caio Raony Farina Silveira, Marcella Cipelli, Carolina Manzine, Silvia Helena Rabelo-Santos, Luiz Carlos Zeferino, Gretel Rodríguez Rodríguez, Josiane Betim de Assis, Suellen Hebster, Isabel Bernadinelli, Fabio Laginha, Enrique Boccardo, Luisa Lina Villa, Lara Termini, Ana Paula Lepique

**Affiliations:** 1 Instituto de Ciências Biomédicas, Universidade de São Paulo, Departamento de Imunologia, São Paulo, Brazil; 2 Faculdade de Farmácia, Instituto de Patologia Tropical e Saúde Pública, Universidade Federal de Goiás, Goiás, Brazil; 3 Universidade Estadual de Campinas, Departamento de Ginecologia e Obstetrícia, Campinas, Brazil; 4 Instituto de Ciências Biomédicas, Universidade de São Paulo, Departamento de Microbiologia, São Paulo, Brazil; 5 Hospital Pérola Byington, São Paulo, Brazil; 6 Departamento de Radiologia e Oncologia, Faculdade de Medicina, Universidade de São Paulo, São Paulo, Brazil; 7 Instituto do Câncer do Estado de São Paulo, São Paulo, Brazil; Instituto Nacional de Cancer, BRAZIL

## Abstract

Cervical cancer, caused by high oncogenic risk Human Papillomavirus (HPV) infection, continues to be a public health problem, mainly in developing countries. Using peptide phage display as a tool to identify potential molecular targets in HPV associated tumors, we identified α-mannosidase, among other enriched sequences. This enzyme is expressed in both tumor and inflammatory compartment of the tumor microenvironment. Several studies in experimental models have shown that its inhibition by swainsonine (SW) led to inhibition of tumor growth and metastasis directly and indirectly, through activation of macrophages and NK cells, promoting anti-tumor activity. Therefore, the aim of this work was to test if swainsonine treatment could modulate anti-tumor immune responses and therefore interfere in HPV associated tumor growth. Validation of our biopanning results showed that cervical tumors, both tumor cells and leukocytes, expressed α-mannosidase. *Ex vivo* experiments with tumor associated macrophages showed that SW could partially modulate macrophage phenotype, decreasing CCL2 secretion and impairing IL-10 and IL-6 upregulation, which prompted us to proceed to *in vivo* tests. However, *in vivo*, SW treatment increased tumor growth. Investigation of the mechanisms leading to this result showed that SW treatment significantly induced the accumulation of myeloid derived suppressor cells in the spleen of tumor bearing mice, which inhibited T cell activation. Our results suggested that SW contributes to cervical cancer progression by favoring proliferation and accumulation of myeloid cells in the spleen, thus exacerbating these tumors systemic effects on the immune system, therefore facilitating tumor growth.

## Introduction

Developing countries continue to be burdened by HPV associated cancer (WHO http://www.who.int/immunization/diseases/hpv/en/). In spite of the available prophylactic vaccines, poor countries still display high rates of cervical cancer and mortality due to this disease. In Brazil, for instance, the National Cancer Agency estimates 16.370 new cervical cancer cases in 2018. Once HPV infection is established, immunization against the virus cannot stop disease progression. Therefore, it is necessary to develop new strategies for clinical intervention, both preventively, in previously untreated patients, as well as patients after treatment with residual disease progression or new tumors. In the present study, we used peptide phage display to search for potential molecular targets in cervical cancer derived cell lines and tumors.

This technique allows for the screening of millions of peptide sequences against a defined target [1]. We have used cervical cancer cell derived cell lines, both *in vitro* and *in vivo* as targets for screening with a commercial peptide phage display library. Among the sequences enriched after 3 screening cycles using HPV positive and negative cervical cancer cell lines or tumors in Nude mice, peptides with similarity to α-mannosidase were identified.

α-mannosidases are a family of enzyme isoforms that are expressed by most cells, among them epithelial cells and macrophages. These enzymes are mainly expressed in the endoplasmic reticulum, ER, and Golgi apparatus, where their activity is related to the synthesis and trimming of glycoproteins, and in the lysosomes, where their activity is related to glycoprotein degradation [2–4]. α-mannosidase deficiency can lead to different outcomes, depending on the deficient isoform. Deficiency in lysosomal α-D-mannosidase activity can cause α-mannosidosis, a syndrome characterized by the accumulation of glycoprotein proteins to be degraded, causing vacuolization in peripheral blood cells and fibroblasts. This leads to different systemic problems as synaptic content release, exocytose and autophagy [2]. Deficiency in α-mannosidase II leads to alterations in N-glycan modified proteins, which display immune stimulatory activity, leading to the development of autoimmune diseases [5]. Swainsonine is a pharmacological inhibitor of α-mannosidase, extracted from locoweed (*Astragalus* and *Oxytropis ssp*). This molecule binds to α-mannosidase regardless of the catalytically active configuration of the enzyme, with inhibition constants in the range of 20 to 50 nM [4,6]. SW inhibits mainly the ER and Golgi apparatus α-mannosidases, but also the lysosomal α-mannosidase [4]. It has been demonstrated that SW treatment inhibits tumor cell migration [7,8], modulates of macrophage activity towards M1 phenotype [9–11] and activates of NK cells [12,13].

Our research group and others have previously reported that HPV associated tumors recruit various leukocyte populations that seem to contribute to the lesion progression and tumor growth [14–17]. α-mannosidase activity in cancer may involve its effect on both tumor cells, through migration and invasion through glycoproteins, as well as on cells in the tumor microenvironment as macrophages and NK cells. Since SW, a well characterized inhibitor of α-mannosidase displays anti-tumor activity, we hypothesized that treatment with swainsonine could inhibit tumor growth either directly or through modulation of tumor associated macrophages, leading to anti-tumor immune responses. We have used a HPV16 positive experimental mouse model to test swainsonine effects on tumor growth and immune responses.

## Materials and methods

### Patient data

For the screening process, we recruited patients with high grade lesions or cervical cancer at the Hospital Pérola Byington with indication for surgical procedure. After biopsy harvesting for diagnosis, a second biopsy within the lesion area (assed by colposcopy) was harvested and immediately frozen and then transferred to Tissue Tek OCT. A total of 10 patients with cancer were enrolled in this study. At the same time, 10 patients with complaints unrelated to HPV associated lesions were enrolled to donate a cervical fragment free of disease. This study was approved by the Ethics Committee of Hospital Pérola Byington (process FR-357104).

For α-mannosidase expression validation, we enrolled a cohort of patients in the Women’s Hospital, State University of Campinas, Brazil, between 2005 and 2011. All participants signed an informed consent formulary at the time of sample before sample use for this study. Ethical approval for this study was granted by the Research Ethics Committee of the State University of Campinas (process #1.647.260). Samples were selected retrospectively, in a completely anonymous way. All samples were collected at least three years before the study. Seventy-six paraffin-embedded cervical tissue specimens including 16 cervicitis, 15 cervical intraepithelial neoplasia grade 2 (CIN2), 15 CIN3, 15 invasive squamous carcinomas and 15 adenocarcinomas were included in the study. All methods were performed in accordance with present guidelines and regulations.

### Cell lines

Cervical cancer derived cell lines were purchased from ATCC. HeLa and SW756 are HPV18 positive, SiHa and CaSki are HPV16 positive and C33A is HPV negative [18–20]. HaCat is an immortalized keratinocyte cell line [21]. TC-1 cell line was kindly donated by Prof. TC Wu, John Hopkins Hospital (Baltimore) and is an epithelial cell line transformed with HPV16 oncogenes E6 and E7 and Ej-ras [22]. All cell lines were maintained in 10% fetal bovine serum in RPMI 1640 (Thermo Scientific, Waltham, MS), buffered with sodium bicarbonate and in 5% CO_2_ atmosphere.

### Biopanning

We used the Ph.D. CXC7 Peptide Phage Display library (New England Biolabs, Ipswich, MA) to screen cervical cancer cells *in vitro*. *In vitro*, we incubated 10^8^ *pfu* of bacteriophages with confluent cultures of SiHa or HeLa cells. Supernatants were discarded after 4 hours incubation and cells were harvested with bound bacteriophages, lysed and lysates used to infect K12 bacteria (provided with the peptide phage display library kit), amplifying the population of bacteriophages that bound to the tumor cells. Four rounds of enrichment were performed with each cell line. By the end of these rounds, we sequenced the bacteriophages to find the enriched peptide sequences. The sequence corresponding to α-mannosidase represented 25% and 22% of the total sequences obtained from SiHa and HeLa cultures, respectively.

### Bacteriophage overlay and detection

Mouse tumors or cervical high grade lesion 5 μm cryosections were stored dehydrated and frozen. When needed, slides were brought to 4°C in a desiccating chamber, and then fixed for 5 min in a solution of 1 part methanol/2 parts acetone. Slides were then dried, rehydrated in PBS, and blocked with 5% fetal bovine serum in PBS for 30 min, room temperature. After discarding the blocking solution, we added 10^7^ *pfu* of the specific or an irrelevant bacteriophage to the sections and incubated for 30 min at room temperature. After washing sections with PBS, we added anti-M13 antibody (Abcam, UK) and incubated for 30 min, at room temperature. After washing the primary antibody, slides were incubated with anti-rabbit secondary antibody and revealed using the Universal Elite Vectastain kit and 3,3’-diaminobenzidine, DAB (Vector Laboratories, Burlingame, CA), following manufacturer’s instructions. Finally, tissue sections were stained with hematoxylin and dehydrated through an ethanol gradient and xylol and mounted with Permount (Sigma-Aldrich, St. Louis, MO). Images were acquired using a BX61 Olympus microscope (Olympus, JP). To quantify the overlay assays, we used Photoshop to reduce magenta, cyan, blue and green color of the stained tissues and highlight yellow to give contrast to the DAB brown color. Following that we used this processed image to quantify the brown labeled area in each image using ImageJ. The data is presented as percentage of labeled area in relation to the total epithelium or stromal area in each image.

### Immunohistochemistry for alpha-mannosidase

Paraffin embedded 4 μm tissue sections were treated with xylol for paraffin removal and then rehydrated through an ethanol gradient from 100% 50% ethanol and 3 washes in phosphate buffered saline, PBS. Blocking and immunohistochemistry was performed using a rabbit polyclonal anti-alpha-1,2 mannosidase (ab51262, Abcam, Cambridge, MA, UK) antibody at 1:150 dilution (antibody previously tittered) in a Ventana Benchmark GX equipment. All other reagentes, buffers, secondary antibody provided by Roche (Ventana Medical Systems, AZ, USA). We followed the manufacturer's recommended protocol for antigen detection. Secondary antibody was a peroxidase conjugate, so that detection of antigen/antibody complex was done through DAB reaction and precipitation. Tissues were counterstained with hematoxylin prior to mounting with Permount (Sigma-Aldrich, St. Louis, MO).

### Western blotting

For western blotting we used lysates from exponentially growing cells. Cell lysates of the indicated cell lines or populations were prepared with RIPA buffer, and 50 μg of protein from each cell line were fractioned in SDS Page gels, transferred to PVDF membranes and probed with anti-α-mannosidase antibody and anti-β-tubulin antibody (GE Healthcare, Chicago, IL). After washing the primary antibody excess, membranes were incubated with anti-rabbit peroxidase conjugated secondary antibody (ThermoFisher Scientific, Waltham, MS) and detection was performed using the ECL plus chemiluminescence kit (GE Healthcare, Chicago, IL). Images were obtained by autoradiography.

### *In vitro* experiments

All cell cultures were maintained or incubated in 10% fetal bovine serum (Cultilab, BR) in RPMI1640 (Thermo Fisher Scientific, Waltham, MS) in a 5% CO_2_ atmosphere incubator, at 37°C. For drug toxicity: TC-1 monolayers were treated with variable concentrations of Swainsonine, SW, (S9263, Sigma-Aldrich, St. Louis, MO.) for 3 days. By the end of the incubation period, cells were harvested and counted to estimate cell numbers and viability, by Trypan blue exclusion. For growth kinetics: 5x10^3^ TC-1, C3, C33A and SW756 cells were seeded 10 cm^2^ plates and incubated them in 2μg/ml SW for up to 3 days, when cells were harvested and counted as described above. For T cell proliferation: lymph node cells were labelled with a vital dye Violet Proliferation Dye 450 (BD Biosciences, Carlsbad, CA) and were seeded 2x10^5^ cells/U bottom well of 96 wells plates and treated with 3 μg/ml SW and or a combination of 10 ng/ml PMA and 1 μg/ml ionomycin (PI). After 4 days incubation, cells were harvested, labelled with anti-CD4 and anti-CD8 antibodies conjugated with fluorophores and analyzed by flow cytometry, using a FACSCanto (BD Biosciences, Carlsbad, CA), where, at least 30.000 events were acquired. For suppression assays: same protocol as for T cell proliferation, except that CD45^+^ tumor associated macrophages, or CD11b^+^ splenocytes were added to the cultures, in ratio of 4 lymphocytes/myeloid cell.

For macrophage phenotype: 0.25 to 0.5x10^6^ macrophages (tumoral or peritoneal) were seeded on 96 wells flat bottom plates and treated with 3 μg/ml SW, and or a combination of 100U/ml IFNγ (Petrotech, Inc., MX) and 10 ng/ml LPS (Sigma-Aldrich, St. Louis, MO) for 3 days. As controls for tumor associated macrophages, we used peritoneal macrophages. By the end of this period, supernatants were harvested for iNOS activity and cytokine secretion determination. The cells were washed once in PBS and lysed in hypotonic buffer containing protease inhibitor cocktail (Sigma-Aldrich, 1:100 dilution) followed by 3 cycles of freezing and thawing. After sedimentation of cell debris, the lysates were used for protein quantification and arginase activity assay as previously described [23]. iNOS activity was measured by the Griess test [24]. Both arginase and iNOS activity data were normalized by protein concentration

For cytokine concentration in the cultures supernatant we used the Cytometric Bead Array Mouse Inflammation Kit (BD Biosciences, Carlsbad, CA) and analyzed by flow cytometry.

### Lectin binding to SW treated cells

Single cell suspensions from C57Black/6 mice bone marrow or spleens were seeded in 28 cm^2^ culture dishes, 2x10^6^ cells/well and immediately treated with 1 or 2 μg/ml SW for 48 hours. TC-1 cells were seeded in 78.5 cm^2^ and 24 hours later, treated with 2 μg/ml SW for 48 hours. All cells were incubated in RPMI supplemented with 10% fetal bovine serum, at 37°C, in a 5% CO_2_ atmosphere. After incubation, cells were harvested and incubated with 0.3 μg/ml biotinylated *Licoperssicum sculentum* lectin (ThermoFisher Scientific, Waltham, MA) (lectin concentration was determined by previous titration with spleen single cell suspensions) for 20 min on ice, washed and then cells were labeled with phycoerythrin conjugated streptavidin (also previously tittered). Cells were then analyzed by flow cytometry, where 50.000 events were acquired.

### *In vivo* experiments

C57Black/6 mice were inoculated with viable 10^5^ TC-1 tumor cells as previously described [16]. Once tumors were detectable by touch, approximately 10 days post injection, we started the intraperitoneal administration of 4mg/Kg SW (Sigma-Aldrich) or PBS (vehicle) for 7 days, daily. Mice we monitored daily and tumor diameters were measured every other day. This protocol was approved by the Ethics Committee on Animal Use at the Institute of Biomedical Sciences (process 008/2015-E).

After euthanasia, tumor, bone marrow, lymph nodes, spleen and resident peritoneal macrophages were harvested for immunophenotyping and T cell activation assays.

### Flow cytometry

Murine tumors were digested with 1mg/ml Collagenase I and IV mixed, at 37°C at 1300 rpm. Lymphoid tissues were mechanically dissociated and erythrocytes from spleens and bone marrow eliminated with hypotonic cell lysis buffer. Single cell suspensions were stained with pre-tittered antibodies and analyzed by flow cytometry, using a FACSCanto II (BD Biosciences). Antibodies used in this work were: Ly6C V450 (clone AL-21), Ly6G PE-Cy7 (clone 1A8), CD3e APC (clone 145-2C11), CD8a PE (clone 53–8.7), CD4 PE-Cy5 (clone RM4-5) from BD Biosciences (Carlsbad, CA), CD45 Alexa-647 (clone 30-F11) from Biolegend (San Diego, CA), CD11b FITC (clone M1/70) from R&D Systems (Minneapolis, MN), F4/80 PE-Cy5 (clone BM8) and Foxp3 Alexa-647 (clone 150D/E4) from eBiosciences (ThermoFisher Scientific, Waltham, MA).

### Cell sorting

CD11b^+^ splenocytes and CD45^+^ population from TC-1 tumors were enriched using positive selection by labeling cells with biotin conjugated anti-CD11b (clone M1/70, BD Biosciences, Carlsbad, CA) and anti-biotin Miltenyi magnetic beads (130-090-485, Miltenyi Biotec, GE) or CD45 Microbeads (130-052-301, Milteny Biotec, GE), respectively. Post sort analysis was performed to evaluate final enrichment of the target population. In general, CD45^+^ cells displayed 95% or more viability and 90% enrichment, and we could enrich splenocytes preparations to approximately 40% CD11b^+^ cells (S1 Fig) and over 95% viable cells. Tumor associated macrophages comprised approximately 85% of the total leukocyte infiltrate in the tumors, CD45^+^ (a representation of these populations in the total tumor suspension can be seen in S1 Fig). The CD45^-^ population includes tumor cells, endothelial cells and fibroblasts.

### Statistical analyzes

Results were represented as average and standard deviation. Data was tested using ANOVA, and p<0.05 was accepted to conclude that differences among groups were significant. For tumor growth kinetics, were used box plots to show experimental variability and compared tumor growth kinetics using the Mann-Whitney U test, accepting p<0.05 to that differences between groups were significant.

## Results

### Cervical tumor and stromal cells express α-mannosidase.

Using a circular 7 amino acid residues peptide phage display library, we identified peptide sequences with binding affinity to HeLa and SiHa cervical cancer cell lines. After 3 rounds of biopanning, we sequenced the DNA of the recovered bacteriophages, and sequences with 71.4 to 100% similarity to α-mannosidase represented 22% and 25% of the sequences recovered from SiHa and HeLa biopannings respectively. α-mannosidases are expressed in the endoplasmic reticulum and lysosomes, where they have a role in glycoprotein synthesis and trimming directly affecting function, or stability [4]. We were particularly interested in the potential role of α-mannosidase on the tumor stroma, due to its expression by macrophages and the data showing that pharmacological blockade of this enzyme with SW can modulate macrophage phenotype [11]. To be noticed, the identified sequence being α-mannosidase means molecules that bind this enzyme, probably substrates should be abundant in the tumor cells.

**Fig 1:**
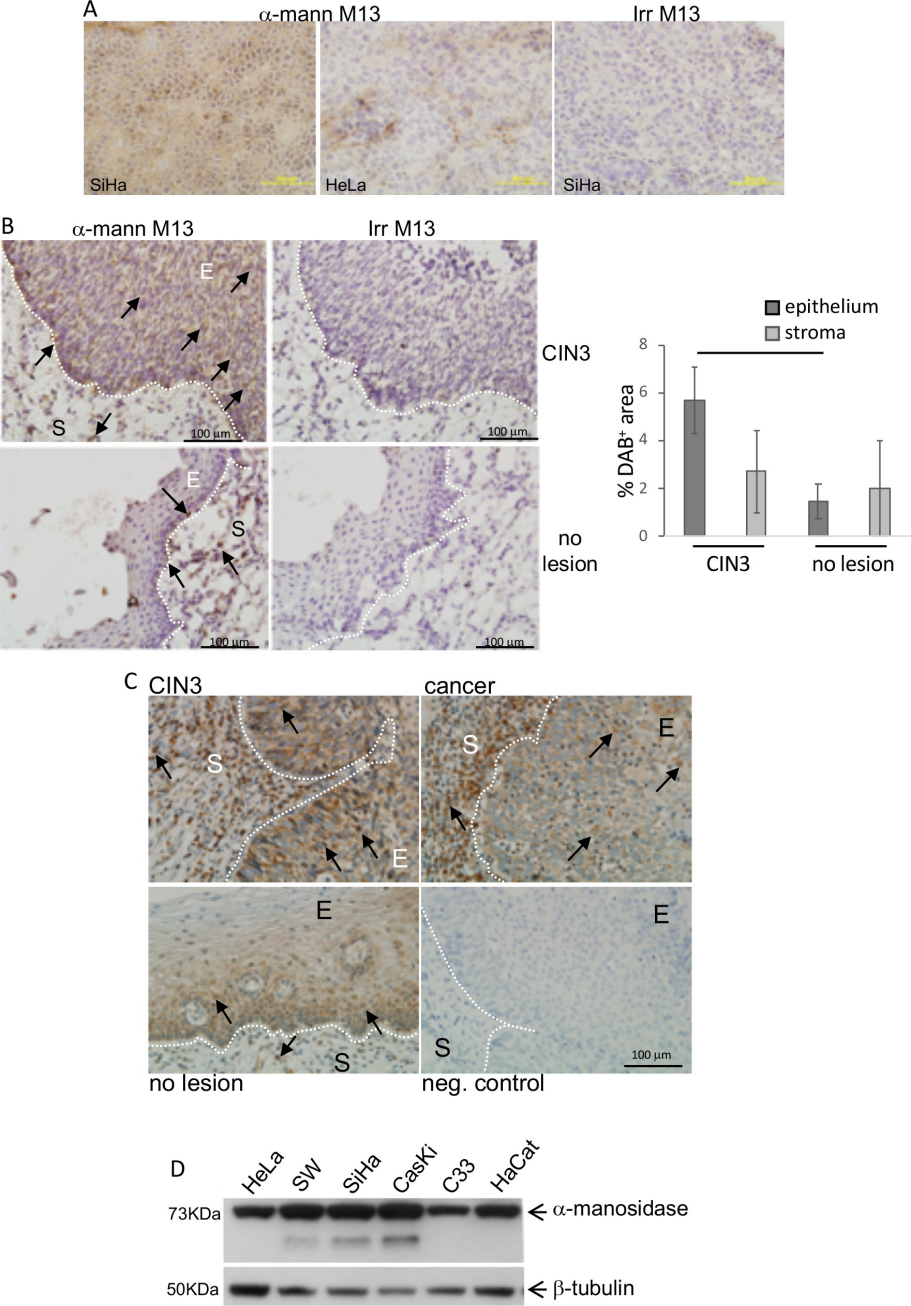
Bacteriophages displaying α-mannosidase peptide preferentially bind to HPV-positive tumor tissue. The validation of our biopanning results was performed on xenografts and human tumor samples. A and B. Overlay assay. The M13 bacteriophage containing the identified α-mannosidase sequence (α-mann M13) or an irrelevant M13 bacteriophage (irr M13) were overlayed on acetone/methanol fixed SiHa and HeLa tumors (A) or in formalin fixed, paraffin embedded high grade cervical intraepithelial neoplasia and control cervical tissue (B, CIN3 and no lesion, respectively). After incubation with 10^7^ pfu M13, tissues were washed, and bound phage detected with anti-M13 antibody and immunohistochemistry. Results are representative of groups of 3 independent tumors in mice, and 10 high grade lesions samples and 7 control samples for cervical tissue. Quantification of the positively labeled areas is represented on the graph on the right. DAB^+^ areas were quantified in the stromal (S) and epithelium (E) compartments of each lesion and compared to the total area of each compartment to result in the percentage of labeled area, which is plotted in the graph. Data was tested by ANOVA; the bar indicates groups with significant differences, p<0.05. C. α-mannosidase expression in high grade cervical lesion, CIN3, invasive cancer (SSC) and control cervical tissue (no lesion). Immunohistochemistry was performed in formalin fixed, paraffin embedded tissues, using anti-α-mannosidase antibody. The negative control corresponds to a cancer sample treated the same way as the other, but without incubation with the primary antibody. Results are representative of 4 invasive cancer samples, 7 high grade lesions and 8 normal samples. In all images, the hatched line limits the stromal (S) and epithelium (E) compartment of each tissue. Arrows indicate examples of DAB stained cells in both stromal and epithelium compartments. D. α-mannosidase expression in cervical cancer derived cell lines and HaCat cells. Arrows indicate the corresponding bands for each specific target protein.

To validate our data, we first performed overlay assays, where we incubated the bacteriophage containing the α-mannosidase peptide or an irrelevant bacteriophage over SiHa and HeLa tumor fixed cryo-sections (Fig 1A). The bacteriophage containing the α-mannosidase sequence bound to the SiHa and HeLa tumors grown in Nude mice (Fig 1A). An irrelevant bacteriophage, on the other hand, was not able to bind to the tumor tissue. We, then, performed the same experiment using high grade cervical lesion sections (CIN3, cervical intraepithelial neoplasia 3) and normal control cervical tissue (Fig 1B). In these tissues, we were able to identify the epithelial and stromal compartments (E and S, Fig 1B), and could estimate the relative binding of the bacteriophage to the tissues by calculating the DAB positive area and total area of each compartment/sample. As can be observed either by the representative images or the quantification of DAB labeled area in Fig 1B, there was significant more bacteriophage binding to the epithelium of tumor samples than control samples. Interestingly, bacteriophage biding to the stromal compartments was not different between tumor and control samples.

Finally, we observed a bacteriophage containing an irrelevant peptide sequence did not bind to either CIN3 or control tissues (Fig 1B, right side image panels).

These results indicated that the bacteriophage isolated from our screening rounds, containing a peptide sequence with similarity to α-mannosidase, had affinity to stromal cells independently of grade lesion, and affinity to epithelial cells in high grade cervical lesions.

We then investigated α-mannosidase expression in cervical lesions and cervical cancer derived cell lines. We observed that cervical tissues expressed α-mannosidase, and that the net expression was higher in high grade lesions and cancer compared to the normal mucosa. This was probably due to the increase in the number of cells expressing the enzyme, including leukocytes present in the tumor stroma, rather than higher protein expression per cell (Fig 1C, S–stromal area). We also investigated the expression of α-mannosidase in cervical cancer derived cell lines, HeLa, SW756, SiHa, CaSki and C33A and in human immortalized keratinocyte cell line, HaCat, by Western blotting. We observed that all cells lines expressed α-mannosidase (Fig 1D). These results suggested that while α-mannosidase may be ubiquitously expressed, the targets that led to the isolation of the peptide related to this enzyme by biopanning seemed to be more abundant in transformed tissue. The presence of molecules that bind to α-mannosidase clearly showed a positive correlation with lesion grade. Therefore, we raised the hypothesis that α-mannosidase activity may be important for cervical tumor progression.

### α-mannosidase inhibitor, Swainsonine, does not block cervical tumor cell proliferation, but modulates macrophage phenotype and activate CD8 T cell proliferation *ex vivo*

Inhibition of α-mannosidase with the pharmacological inhibitor, Swainsonine (SW), has been shown to cause anti-tumor effects, either by inhibiting metastasis or tumor growth itself [7,25,26]. Given our previous data, we decided to test whether the use of SW could inhibit cervical cancer cells proliferation and tumor growth. Our laboratory has been working with the TC-1 experimental tumor model for many years, and the greatest advantage of this cell line, besides expressing HPV16 E6 and E7, is that it is isogenic to C57Black/6 mice and therefore can be tested in an immunocompetent context.

**Fig 2:**
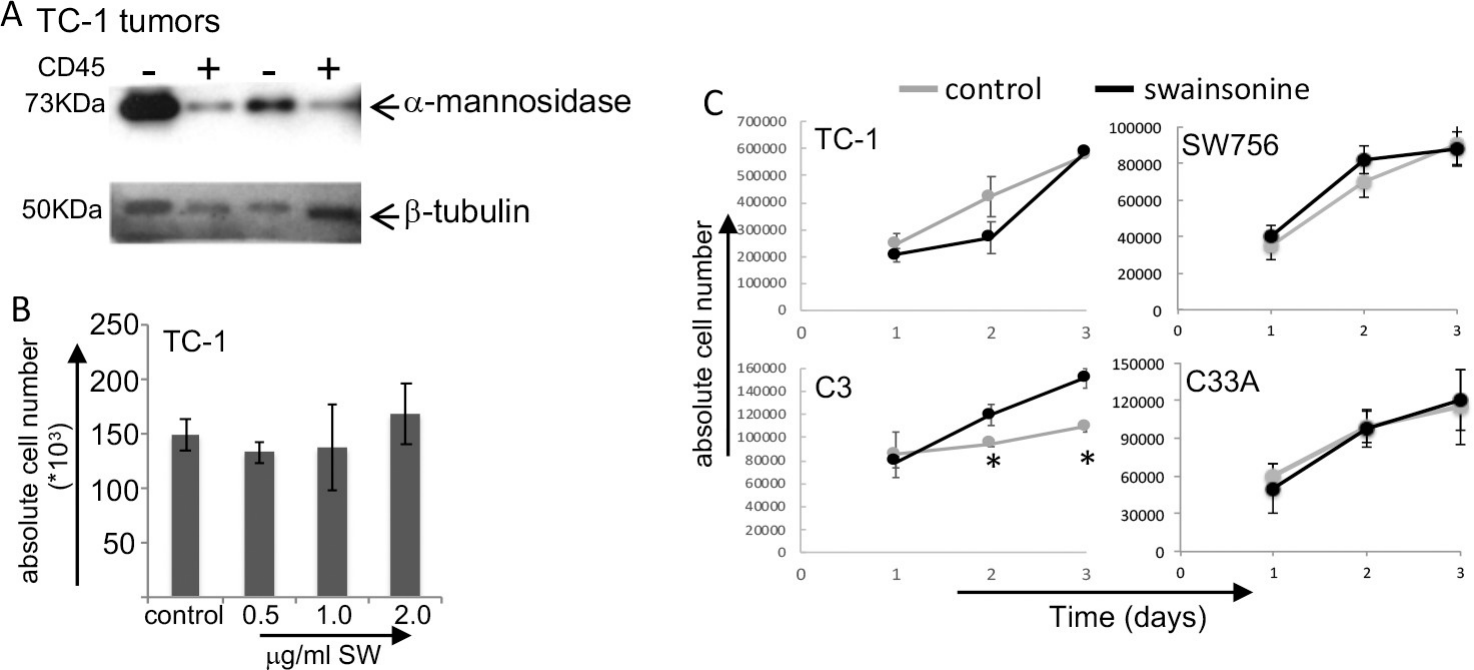
Swainsonine treatment does not inhibit tumor cell proliferation in vitro. A. TC-1 tumors single cell suspensions were sorted into CD45^+^ and CD45^-^ populations for lysis and detection of α-mannosidase and β-tubulin as control, by Western blotting. B. Dose-response curve of TC-1 cells treated for 3 days with the SW indicated concentrations. C. Cell growth kinetics of murine HPV transformed cells lines, TC-1 and C3, and cervical cancer derived cell lines, SW756 and C33A. Cells were treated with 2μg/ml SW for the indicated periods. In both B and C, absolute cell numbers were determined by cell counting aliquots of cell suspensions in a Neubauer chamber under the microscope. Cell viability was determined by Trypan blue exclusion. In all cases, the percentage of dead cells was < 5%. Data results from 3 independent experiments with triplicates in each case.

We first harvested TC-1 tumors and then sorted CD45^+^ cells from the rest of the tumor cells and evaluated the expression of α-mannosidase in both compartments. Sorting of CD45^+^ population isolates leukocytes from other cell populations, CD45^-^, as tumor cells and endothelial cells. As shown in Fig 2A, both the CD45^-^ and CD45^+^ cells express α-mannosidase *in vivo*. This result concurs with the previous observation that cells in the stroma of cervical tumors expressed α-mannosidase. We proceeded to test if the α-mannosidase inhibitor, SW could interfere in tumor cell proliferation *in vitro*. We detected no alteration in the viability or proliferation of TC-1 cells *in vitro* (Fig 2B and Fig 2C). Interestingly, SW treatment did not change proliferation of human cervical cancer derived cell lines SW756 (HPV18 positive) and C33A (HPV negative), however, it increased the proliferation of the C3 murine tumor cell line (Fig 2C). The main difference between the C3 and TC-1 cell lines is that the first contains a plasmid with all HPV16 genes cloned, and the second a retrovirus that expresses only HPV16 E6 and E7. We expect E6 and E7 expression levels should be different between these cell lines, higher in TC-1. However, how exactly this could influence the *in vitro* response to SW is still to be investigated. Finally, we observed that SW treatment increased the biding of lectin to the surface of TC-1 cells (S2 Fig). Both the percentage of lectin positive cells increased as the binding per cell (measure by median fluorescence intensity, MFI) increased upon treatment, indicating that treatment altered the composition of glycoproteins on the cell surface.

**Fig 3:**
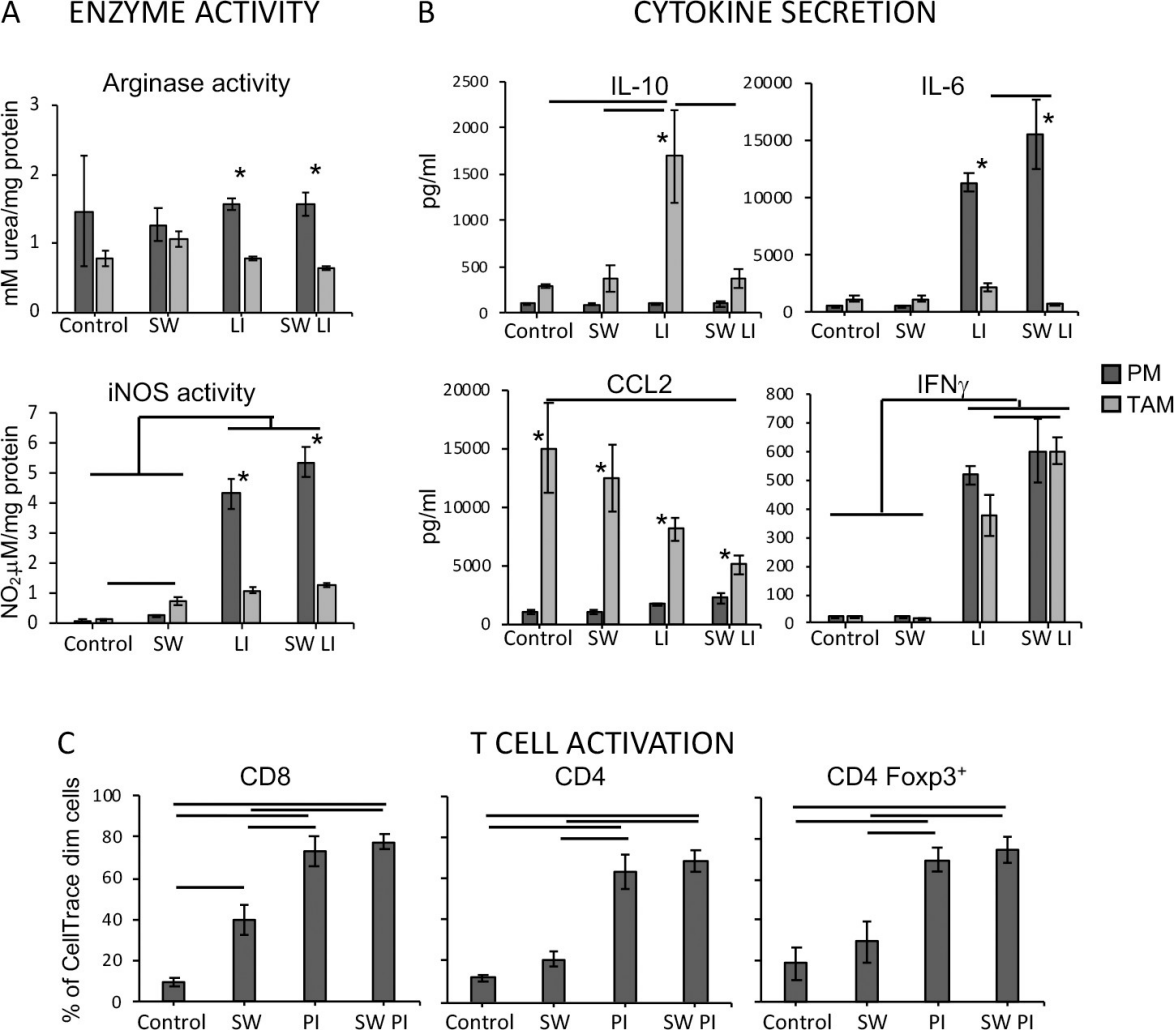
Swainsonine partially modulates tumor-associated macrophages and activates CD8 T in vitro. A. Arginase and iNOS activities detected in lysates and supernatants, respectively, from peritoneal macrophages (PM) or tumor associated macrophages (TAM). PM from the peritoneal lavage and TAM sorted from TC-1 tumors were stimulated for 3 days with 2 μg/mL SW and or combination of 100U/mL IFNγ and 10 ng/mL LPS (LI). B. Cytokine concentration measured in the supernatants of the cultures described above, using the CBA Inflammatory cytokine kit (BD Biosciences) and flow cytometry. C. T cell proliferation assay. Lymph node cell suspensions were labeled with Cell Dye and cultured for 4 days in the presence of 2μg/ml SW, or 10ng/ml PMA and 1μg/ml Ionomycin (PI) or a combination of all these factors. All experiments were performed in triplicates and repeated at least 3 times. The graphs show the averages and standard deviation of all samples tested. Significant differences are indicated by * between PM and TAM or bars among the different treatments.

Most of the CD45^+^ cells in the TC-1 tumors leukocyte infiltrate are macrophages [15] (S1 Fig). We therefore isolated the tumor associated macrophages, TAM, to test if inhibition of α-mannosidase could modulate their phenotype. Comparing these macrophages with peritoneal resident macrophages (PM), we observed that SW had no effect, either alone or in combination with LPS and IFNγ treatment, on the Arginase activity. All treatments led to a slight but significant increase in iNOS activity in TAM. Regarding cytokine production, we observed that combined treatment with LPS and IFNγ (LI) led to a significant increase in IL-10 secretion by TAM, which was counteracted by SW. The same was observed with IL-6, LI induced IL-6 expression in TAM, but the co-treatment with SW counteracted its effect (bar indicates the difference between TAM treated with LI and LI plus SW). We also observed that CCL2, which was secreted by TAM, as previously described [16], displayed significantly lower concentration when cells were treated with a combination of SW and LI. CCL2 is important for monocyte recruitment and modulation of macrophages towards M2 [14,27]. Finally, we observed that LI treatment induced IFNγ expression on both peritoneal macrophages and TAM (Fig 3B). These results indicated that SW was not a strong modulator of macrophage phenotype, but could suppress some important features of TAM, as activation induced IL-10 and CCL2 secretion [15]. Data in the literature shows that alterations in mannose residues in the T cell receptor complex, TCR, caused by SW treatment can increase T cell activation [7,8,28]. We therefore tested the effect of SW treatment on T cell proliferation. We observed that CD8 proliferation increased significantly in response to SW, while CD4 and regulatory T cell proliferation rates were unchanged by this treatment. In all cases, non-specific stimulus with PMA and ionomycin induced robust T cell proliferation (Fig 3C and S3 Fig).

### Swainsonine treatment increased tumor growth through the increase of immunossupression

**Fig 4:**
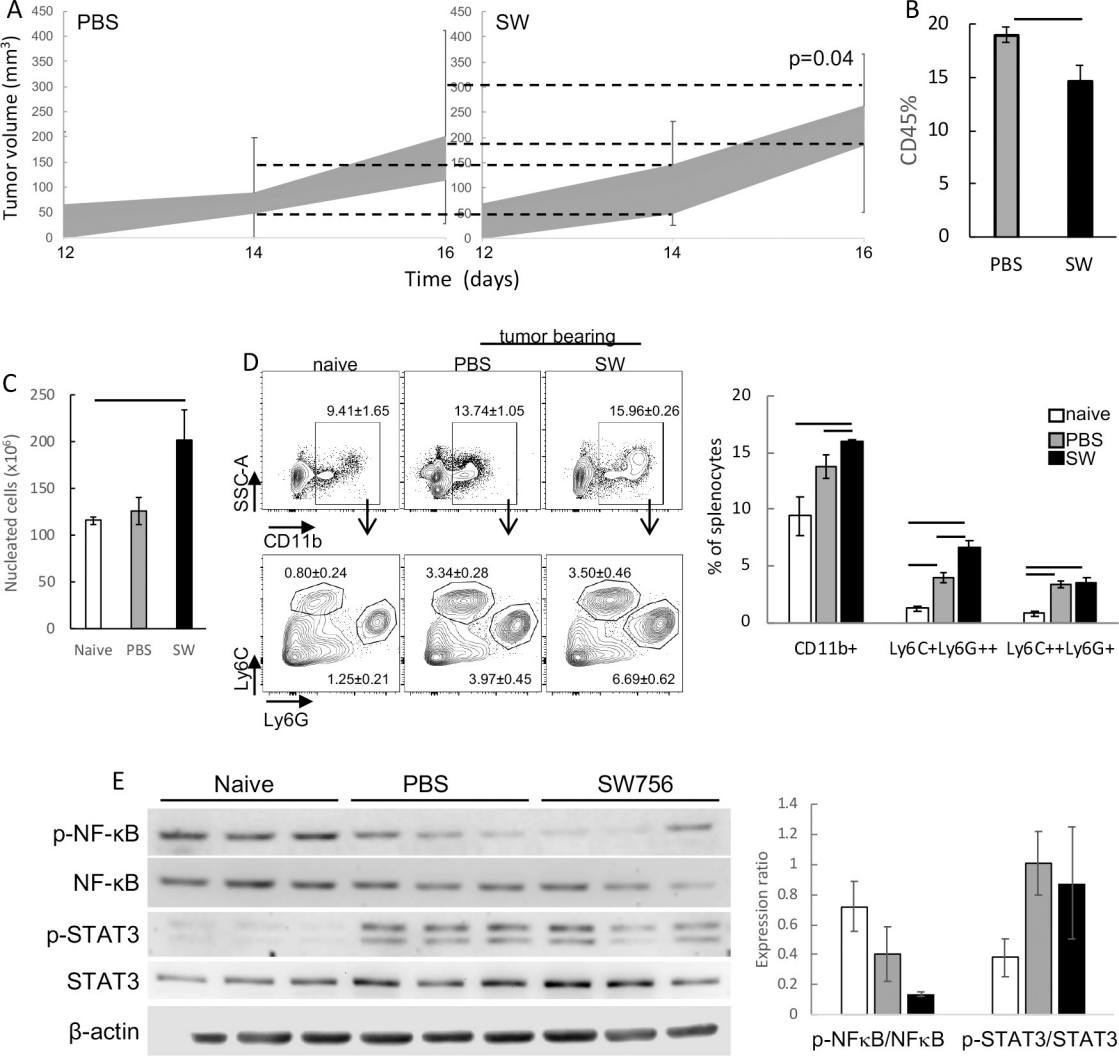
Treatment of mice with SW enhances tumor growth and increase spleen myeloid populations. A. Tumor growth kinetics. C57Black/6 mice were injected with 10^5^ TC-1 tumor cells and treated daily with 4 mg/Kg SW or PBS for 7 consecutive days after tumors detection. Data is represented in box-plots and dashed lines indicate the variation in tumor growth at days 14 and 21 post tumor cell injection between SW and PBS treated groups. Results were compared by Mann-Whitney U test, p value indicated. B. Frequency of tumor infiltrating leukocytes, CD45^+^ cells, detected by flow cytometry after tumor harvesting, digestion with Collagenase and labelling with anti-CD45. C. Spleen cellularity in control mice (naive) and tumor bearing mice (PBS or SW treated). D. Frequency of myeloid spleen populations in the experimental groups described above. Single cell spleen suspensions were labeled with antibodies against the indicated markers and analyzed by flow cytometry. The Ly6C and Ly6G subpopulations (inferior plots) were gated on the CD11b^+^ cells (superior plots). Quantification of the different highlighted populations in the dot-plots is shown in the graph at the right side. Bars indicate significant differences among experimental groups. E. Western blotting showing the expression of phospho (P) and total NFκB and STAT3 in spleen lysates. β-actin was used as loading control. Spleen preparations from 3 different mice per group were used for this experiment. The graph to the right shows expression ratios calculated as follows: densitometric value of each band was divided by the respective β-actin densitometry value to normalize the expression of each of the target proteins; normalized values of p-NFκB were divided by normalized values of NFκB, and the same was done with STAT3. These results are represented in the graph.

The results described above prompted us to investigate whether SW and may have any effect on tumor growth, mainly through the modulation of the tumor microenvironment. We have previously shown that TAM can inhibit anti-tumor T cell responses. Therefore, our rationale was that the combination of CD8 potential activation, together with modulation of macrophage phenotype could inhibit tumor growth. To test this hypothesis, we used a well characterized murine tumor model and treated mice with SW to observe tumor growth. Upon treatment, we were surprised to observe that tumors in treated mice grew significantly faster than tumors in control mice (Fig 4A). Interestingly, there was a significant inhibition in the recruitment of cells to the tumor microenvironment in animals treated with SW. There an increase in 30% in the frequency of CD45^+^ cells in control tumors than in tumors from SW treated mice (Fig 4B). Although we have not accessed the concentration of CCL2 in SW treated animals, this result corroborates the observation that SW may decrease CCL2 secretion by TAM, reducing the recruitment of cells to the tumor microenvironment.

Ours and other research groups have previously shown that TC-1 tumors, as cervical tumors in patients, display systemic effects on cells of the immune system [16,17,29]. Here we observed significant differences in the myeloid populations in the spleen of treated mice. Comparing SW treated tumor bearing mice with the other experimental groups, naïve controls or tumor bearing PBS treated mice, we observed that mice in the SW group displayed significant higher spleen cellularity than the others (Fig 4C). Regarding myeloid populations, both tumor bearing groups had significant higher frequencies of CD11b^+^Ly6C^+^Ly6G^+^ cells in the spleen than naïve controls. Between SW and PBS treated tumor bearing mice, we observed that SW treated mice had significant higher frequency of CD11b^+^Ly6C^+^Ly6G^++^ splenocytes (Fig 4D). These myeloid populations have been described in the literature as monocytic or granulocytic myeloid-derived suppressor cells [30]. In mice with tumors, these cells have been shown to be responsible for suppression of anti-tumor T cell responses. Importantly, in naïve mice, SW treatment decreased the overall frequency of CD11b^+^ cells, but no changes in Ly6C and Ly6G subpopulations were observed (S3 Fig). This result indicated that, in tumor bearing mice, SW added to the systemic effects triggered by the tumor but had no effect by itself.

STAT3 and NFκB activities have been associated with different leukocyte phenotypes and anti-tumor responses [31]. Further characterization of these animals showed that there was higher expression of phospho-STAT3 in the splenocytes of tumor bearing mice compared to naïve, independently of the SW treatment. On the other hand, expression of phospho-p65 NFκB in the splenocytes was lower in SW treated tumor bearing mice, than in control tumor bearing mice and even lower than in splenocytes of naïve mice (Fig 4E). These results corroborated the hypothesis that SW treatment contributed to the accumulation of myeloid-derived cells in the spleens of tumor bearing mice. In order to test this hypothesis, we performed co-culture assays with CD11b^+^ enriched splenocytes from tumor bearing mice and activated T cells from control mice.

**Fig 5:**
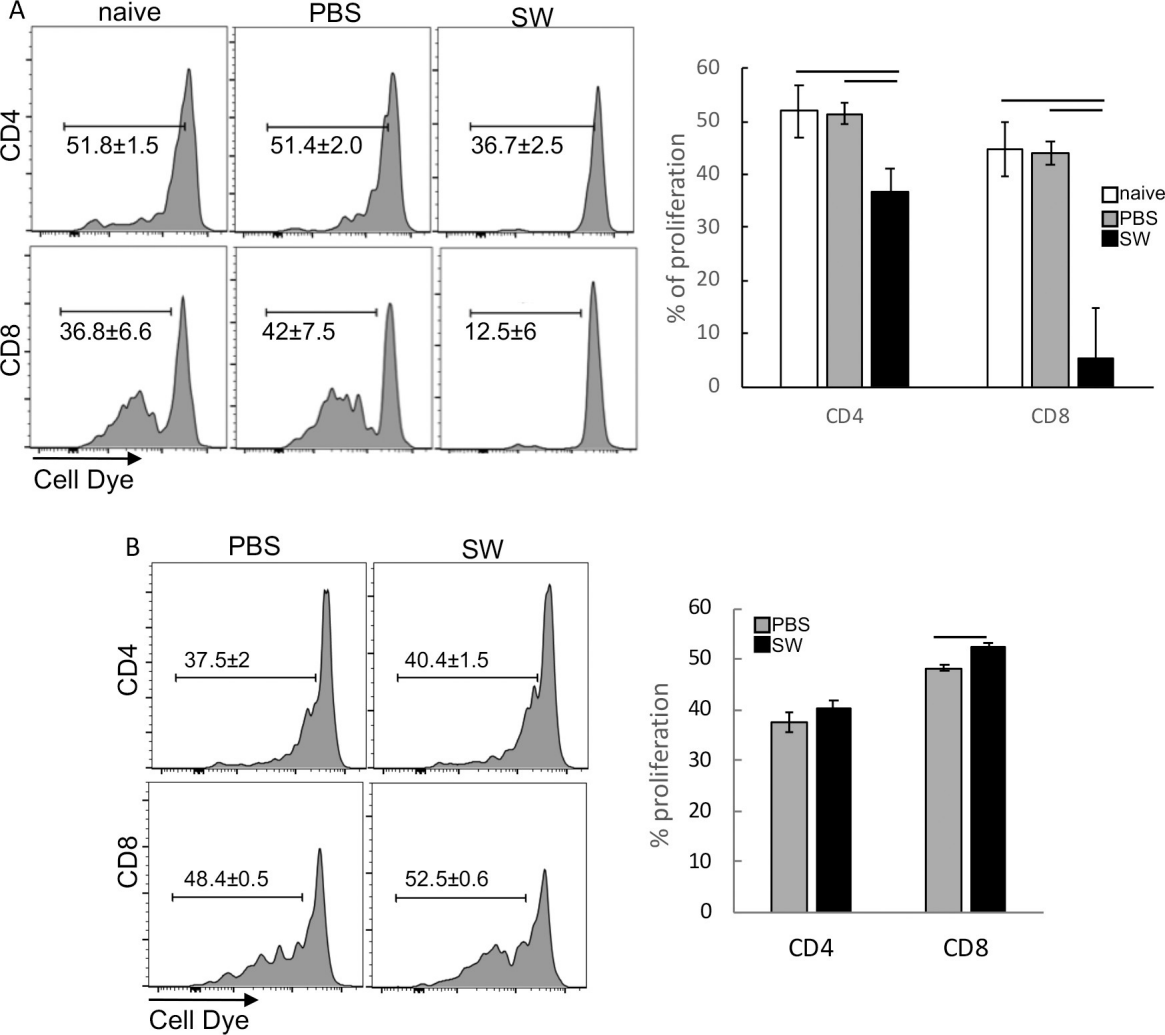
SW treatment further promotes suppression of T cells by MDSC. A. T cell proliferation suppression assay with spleen CD11b^+^ cells of tumor-bearing mice. Enriched CD11b^+^ splenocytes from naive or tumor bearing mice treated with SW or PBS were co-cultured for 4 days with previously labelled and stimulated naive T cells, in a 1:4 ratio. T cells were labelled with Cell Dye and stimulated with 10ng/ml PMA and 1μg/ml Ionomycin (PI). After harvesting, cells were labelled with anti-CD4 and anti-CD8 and analyzed by flow cytometry, using a FACSCAnto, where, at least 10.000 events were acquired. B. Same assay using splenocytes from SW treated naïve mice. A total of 3 mice were analyzed per experiment. Results were tested by ANOVA; bars indicate the groups among which we observed significant differences where p<0.05.

We observed that splenocytes from the SW treated group significantly inhibited T cell proliferation compared to the other groups (Fig 5A). Importantly, splenocytes from naïve mice treated with SW increased CD8 T cell proliferation, instead of inhibiting it (Fig 5B). These results indicated that SW, by itself, did not inhibit T cells, but potentiated the suppressive environment caused by TC-1 tumors, by increasing the population of myeloid suppressor cells.

As observed with TC-1 cells, SW treatment caused changes on the surface glycoproteins of bone marrow cells and splenocytes. This was indicated by the dose-response increase in the binding of tomato lectin to bone marrow and spleen single cell suspensions upon SW treatment for 48 hours (S2 Fig).

## Discussion

Using the peptide phage display method, we identified heptapeptide sequences with preferential affinity to cervical cancer cell lines and tumors. One of the sequences corresponded to α-mannosidase, which indicated that substrates of this enzyme should be abundant in these tumors. Interestingly, the overlay validation tests showed that the bacteriophage containing the α-mannosidase corresponding peptide displayed stronger binding to cervical tumor tissue than to normal cervical epithelium, again indicating that targets of this enzyme are more abundant in the tumor tissue. α-mannosidase aberrant activity, as well as the activity of other enzymes involved in protein glycosylation, has been associated with cancer progression due to the accumulation of N-glycans on the cell surface [32–34]. In line with this observation, the use of a plant derived α-mannosidase inhibitor, SW, has been shown to inhibit tumor growth and metastasis in experimental models [35] and induce tumor mass shrinkage in clinical trials [36].

In our model, we observed that α-mannosidase was expressed both by normal and cancer tissue and by the immortalized human keratinocyte cell line, HaCat, and cervical cancer derived cell lines. However, the net mannosidase expression in tumor tissue was higher than in normal tissue. This was not only due to the abundance of tumor cells, but also a consequence of the recruitment and accumulation of leukocytes in the tumor stroma, which were also positive to α-mannosidase. This observation was particularly interesting to our laboratory, since we have been investigating the tumor microenvironment in cervical cancer [17]. Moreover, data in the literature showed that SW can activate cells from the immune system, including macrophages, NK cells and CD8 T cells. Our data corroborated these observations, since we showed that SW treatment could directly activate CD8 T cells from naïve mice and could partially modulate tumor associated macrophages from a HPV16 associated tumor model, TC-1. One of the interesting observations was that SW could reduce the secretion of CCL2 by TAM. We have reported that TAM express more CCL2 than tumor cells [16]. In addition, Pahler and collaborators have shown the importance of this chemokine in the recruitment of monocytes to the tumor microenvironment [14]. Moreover, SW could counteract the stimulatory effects of IFNγ and LPS treatment on the induction of IL-10 and IL-6 secretion by TAM. Both these cytokines have been associated to the suppression of T cell responses [16,37,38]. These results prompted us to test SW effect on TC-1 tumor growth *in vivo*. To our surprise, tumor growth was significantly increased in mice treated with SW. Interestingly, SW effect on tumor growth seemed to be indirect, through the increase in the frequency of MDSC in the spleen of tumor bearing mice.

Cervical tumors, the best characterized HPV associated tumors, display systemic effects on the immune system, these are triggered, for example, by cytokines as G-CSF that promote leukocytosis by signaling through STAT3 [29,39]. We have previously described, in experimental tumor models, an increase in myeloid cell proliferation in the bone marrow and spleen of tumor bearing mice compared to controls [16,40]. Moreover, we have shown that patients with cervical intraepithelial neoplasia exhibited an increase in the frequency of circulating CD66b^+^ low density neutrophils, again indicating systemic effects of these lesions, even before progressing to cancer [17]. Besides HPV associated cancers, other types of solid tumors can induce leukocytosis, including pancreas, non-small lung and colorectal cancer [41–43]. Leukocytosis can lead to the accumulation of MDSC that inhibit T cell responses, and by extension hamper anti-tumor cell cytotoxic activity.

Although we observed no effect of SW on myeloid cells of naïve mice, it clearly contributed to the systemic effects of the tumor on the immune system of tumor bearing treated mice. Data in the literature have shown that SW can promote cell proliferation in the bone marrow, protecting mice from doxorubicin treatment [44]. In mice bearing B16 melanoma tumors, SW could protect the bone marrow against cyclophosphamide treatment, without interfering with the efficacy of treatment against the tumor [45]. Importantly, in this study, SW treatment alone had no effect on B16 tumor growth. Although B16 tumors recruit TAM to the microenvironment, they are not as robust in inducing leukocytosis as HPV associated tumors [46].

Immune responses are important in the control of HPV associated lesions, as highlighted by the fact that patients with acquired immunodeficiencies, either through HIV infection or iatrogenic, are at higher risk of developing HPV associated cancers. Although HPV associated tumors still burdens women and men, mainly is developing countries and therapeutic solutions are important to the population of infected people, our data suggests that SW would be not be a drug of choice for these potential patients. The exacerbation of immune suppression through the increase and accumulation of MDSC would work to promote tumor progression and growth instead of protection.

## Supporting information

S1 Fig Enrichment of CD11b^+^ splenocytes and macrophage gating strategy. A. Enrichment of CD11b^+^ splenocytes. Left side, example of pre and post sort. Right side. Average enrichment of CD11b^+^ cells from spleens of each experimental group. B. Gating strategy to identify TAM. After harvesting, tumors were digested with 1mg/ml Collagenase I and IV and single cell suspensions were labeled with previously tittered antibodies against CD45, CD11b and F4/80. Cells were analyzed by flow cytometry using a FACSCanto II, were at least 50.000 events were acquired. After exclusion of debris and doublets, we gated on the CD45^+^ population to identify the macrophage population, CD11b^+^F4/80^+^, which constitutes the vast majority of inflammatory cells in the tumor.
(PDF)

S2 Fig SW treatment changes lectin binding to the surface of tumor cells and leukocytes. A. TC-1 cells and bone marrow and spleen single cell suspensions were treated with 1 or 2 μg/ml SW for 48 hours, before harvesting. Cells were then incubated with 0.3 μg/ml biotinylated tomato lectin, washed and then incubated with phycoerythrin conjugated streptavidin. Cells were analyzed by flow cytometry. Only one experiment was performed. Dose-response effect on splenocytes and bone marrow cells are indicative of the reproducibility of the results. B. Representative flow cytometry dot-plots of lectin binding to splenocytes. Plots were obtained after debris and doublets exclusion. No lectin–cells incubated only with streptavidin; untreated control–basal lectin binding to untreated cells, 1 and 2 μg/ml SW–cells treated with SW and then labeled with lectin.
(PDF)

S3 Fig Example of T cell proliferation assays and frequency of myeloid cells in SW treated naïve mice. A. Example of T cell proliferation assay. Cell Dye labeled T cells were incubated with 10 ng/ml PMA and 1 μg/ml Ionomycin for 4 days, harvested, labeled with anti-CD4 and anti-CD8 and analyzed by flow cytometry. B. Frequency of myeloid cells in the spleens of naïve mice treated with PBS or 4 mg/Kg SW for 7 days. Ly6C and L6G cells are also CD11b^+^. * indicates significant difference between experimental groups.
(PDF)
