# Supplementary material for: Swainsonine, an alpha-mannosidase inhibitor, may worsen cervical cancer progression through the increase in myeloid derived suppressor cells population
Source: PLoS One. 2019 Mar 6;14(3):e0213184. doi: 10.1371/journal.pone.0213184 (PMC6402676; doi:10.1371/journal.pone.0213184)
Supplement: S1 Fig — A. Enrichment of CD11b+ splenocytes. Left side, example of pre and post sort. Right side. Average enrichment of CD11b+ cells from spleens of each experimental group. B. Gating strategy to identify TAM. After harvesting, tumors were digested with 1mg/ml Collagenase I and IV and single cell suspensions were labeled with previously tittered antibodies against CD45, CD11b and F4/80. Cells were analyzed by flow cytometry using a FACSCanto II, were at least 50.000 events were acquired. After exclusion of debris and doublets, we gated on the CD45+ population to identify the macrophage population, CD11b+F4/80+, which constitutes the vast majority of inflammatory cells in the tumor. (PDF) [file pone.0213184.s001.pdf]

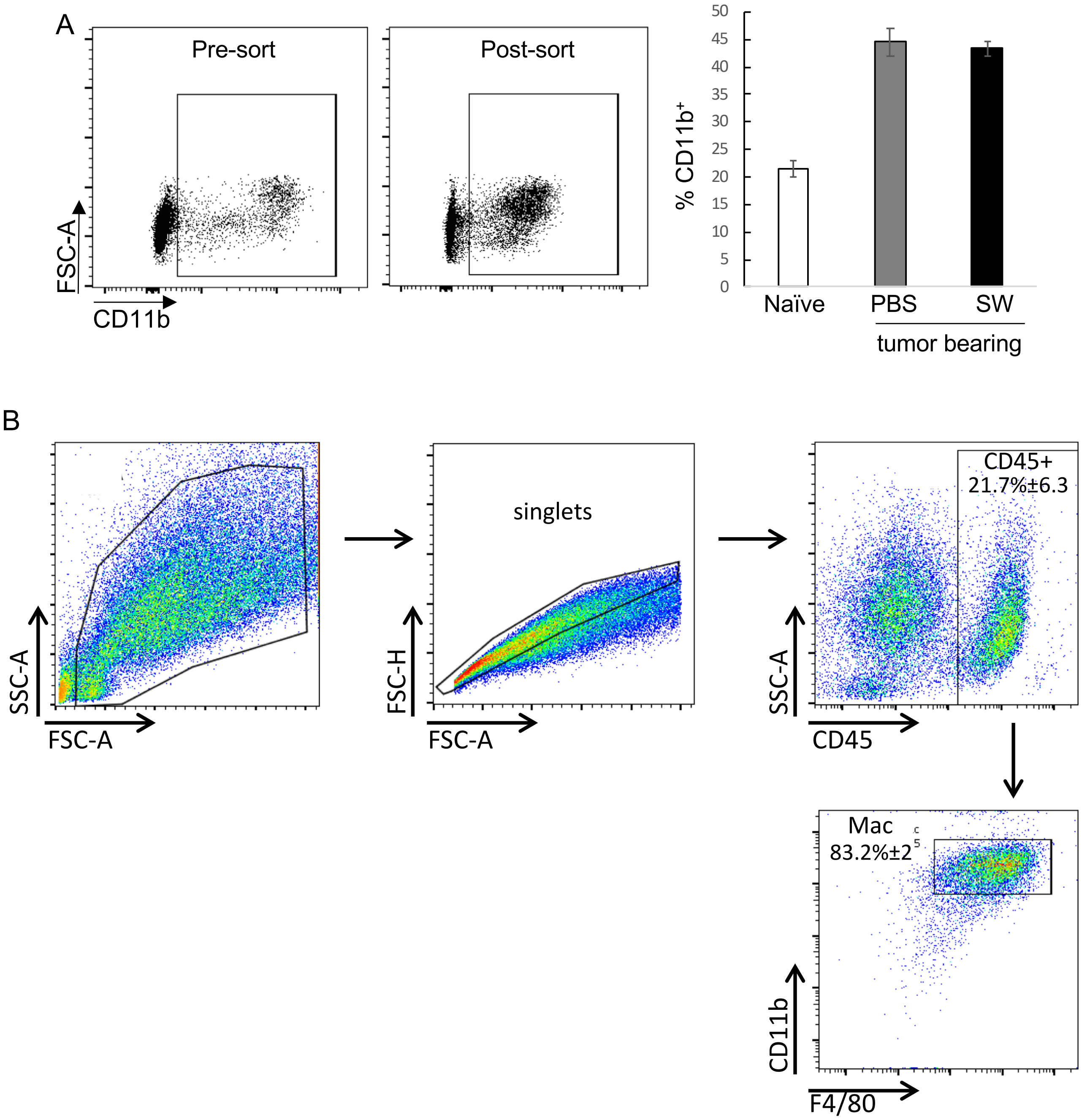

S1 Fig. Enrichment of CD11b+ splenocytes and macrophage gating strategy.

A. Enrichment of $CD11b^+$ splenocytes. Left side, example of pre and post sort. Right side. Average enrichment of $CD11b^+$ cells from spleens of each experimental group. B. Gating strategy to identify TAM. After harvesting, tumors were digested with 1mg/ml Collagenase I and IV and single cell suspensions were labeled with previously tittered antibodies against CD45, CD11b and F4/80. Cells were analyzed by flow cytometry using a FACSCanto II, were at least 50.000 events were acquired. After exclusion of debris and doublets, we gated on the CD45+ population to identify the macrophage population, CD11b+F4/80+, which constitutes the vast majority of inflammatory cells in the tumor.
