# Supplementary material for: Swainsonine, an alpha-mannosidase inhibitor, may worsen cervical cancer progression through the increase in myeloid derived suppressor cells population
Source: PLoS One. 2019 Mar 6;14(3):e0213184. doi: 10.1371/journal.pone.0213184 (PMC6402676; doi:10.1371/journal.pone.0213184)
Supplement: S2 Fig — A. TC-1 cells and bone marrow and spleen single cell suspensions were treated with 1 or 2 μg/ml SW for 48 hours, before harvesting. Cells were then incubated with 0.3 μg/ml biotinylated tomato lectin, washed and then incubated with phycoerythrin conjugated streptavidin. Cells were analyzed by flow cytometry. Only one experiment was performed. Dose-response effect on splenocytes and bone marrow cells are indicative of the reproducibility of the results. B. Representative flow cytometry dot-plots of lectin binding to splenocytes. Plots were obtained after debris and doublets exclusion. No lectin–cells incubated only with streptavidin; untreated control–basal lectin binding to untreated cells, 1 and 2 μg/ml SW–cells treated with SW and then labeled with lectin. (PDF) [file pone.0213184.s002.pdf]

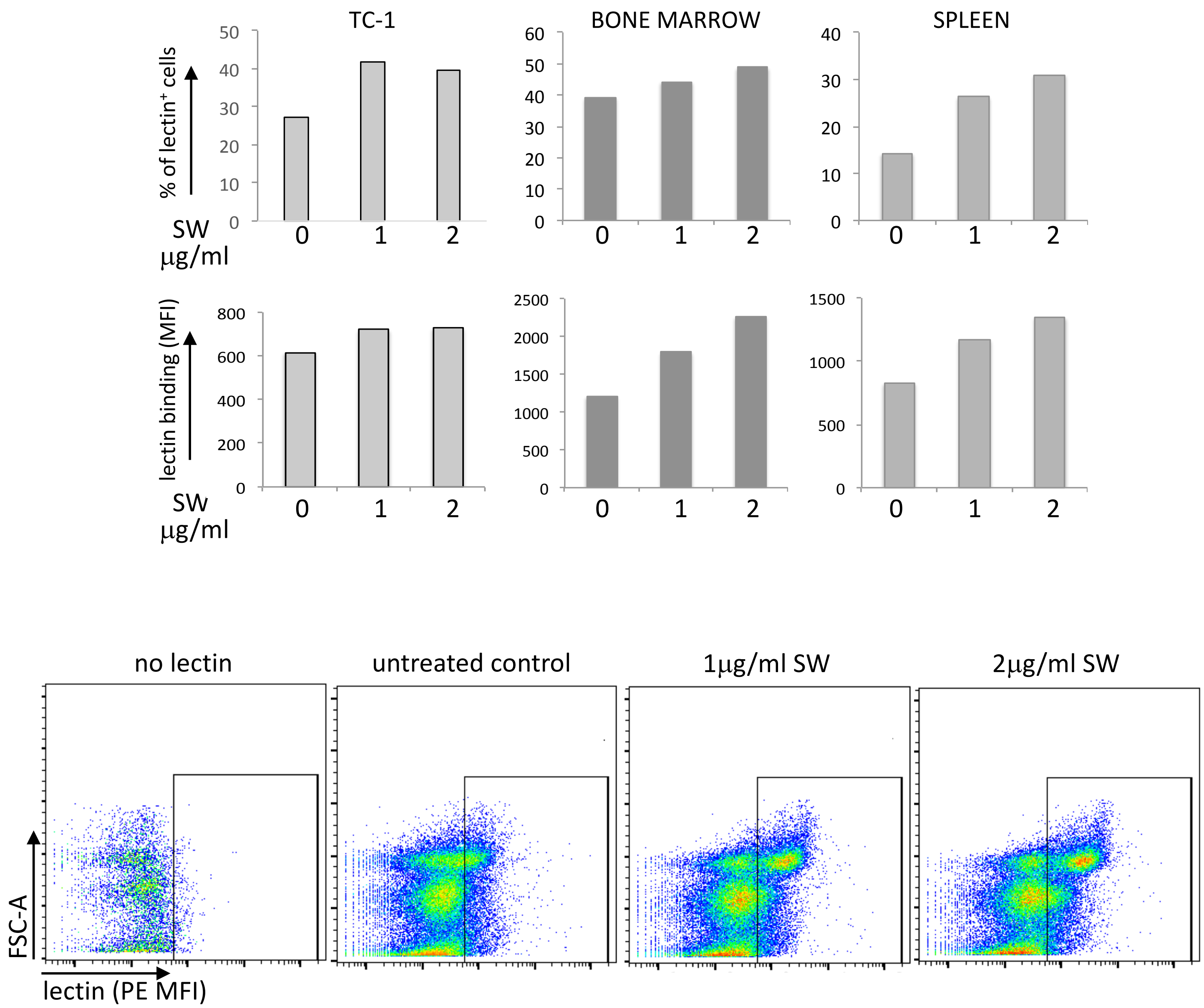


S2 Fig. SW treatment changes lectin binding to the surface of tumor cells and leukocytes. A. TC-1 cells and bone marrow and spleen single cell suspensions were treated with 1 or 2 µg/ml SW for 48 hours, before harvesting. Cells were then incubated with 0.3 mg/ml biotinylated tomato lectin, washed and then incubated with phycoeritrin conjugated streptavidin. Cells were analyzed by flow cytometry. Only one experiment was performed. Dose-response effect on splenocytes and bone marrow cells are indicative of the reproducibility of the results. B. Representative flow cytometry dot-plots of lectin binding to splenocytes. Plots were obtained after debri and doublets exclusion. No lectin – cells incubated only with streptavidin; untreated control – basal lectin binding to untreated cells, 1 and 2 µg/ml SW – cells treated with SW and then labeled with lectin.
