# Supplementary material for: Swainsonine, an alpha-mannosidase inhibitor, may worsen cervical cancer progression through the increase in myeloid derived suppressor cells population
Source: PLoS One. 2019 Mar 6;14(3):e0213184. doi: 10.1371/journal.pone.0213184 (PMC6402676; doi:10.1371/journal.pone.0213184)
Supplement: S3 Fig — A. Example of T cell proliferation assay. Cell Dye labeled T cells were incubated with 10 ng/ml PMA and 1 μg/ml Ionomycin for 4 days, harvested, labeled with anti-CD4 and anti-CD8 and analyzed by flow cytometry. B. Frequency of myeloid cells in the spleens of naïve mice treated with PBS or 4 mg/Kg SW for 7 days. Ly6C and L6G cells are also CD11b+. * indicates significant difference between experimental groups. (PDF) [file pone.0213184.s003.pdf]

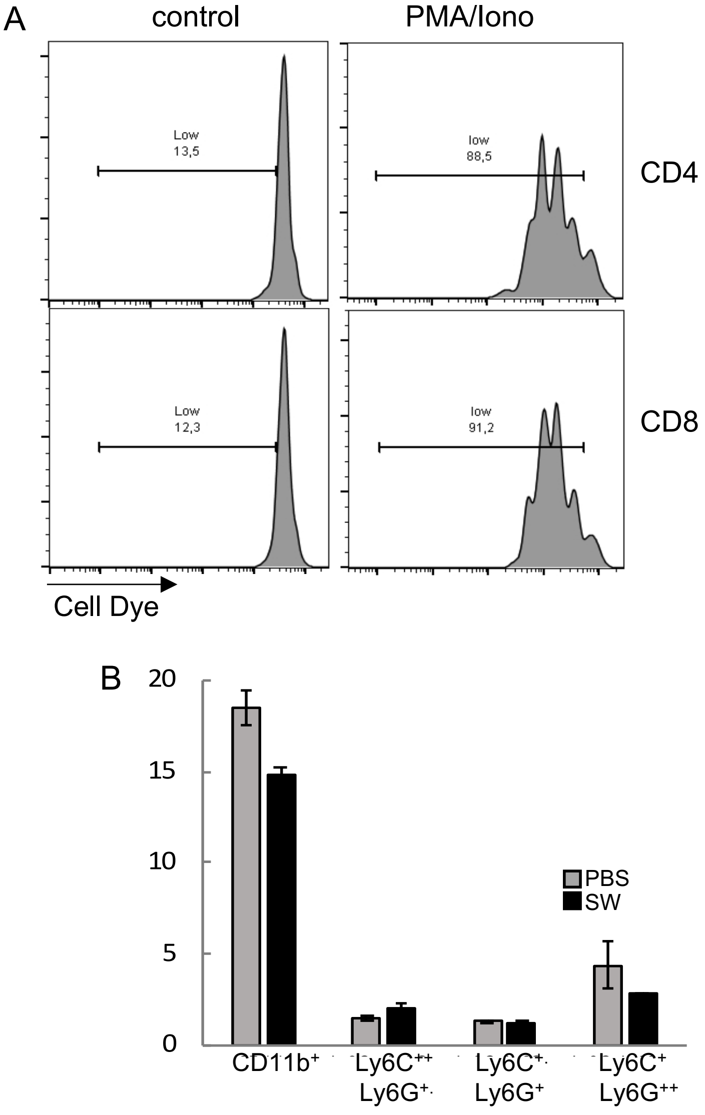

S3 Fig. Example of T cell proliferation assays and frequency of myeloid cells in SW treated naïve mice. A. Example of T cell proliferation assay. Cell Dye labeled T cells were incubated with 10 ng/ml PMA and 1 μg/ml Ionomycin for 4 days, harvested, labeled with anti-CD4 and anti-CD8 and analyzed by flow cytometry. B. Frequency of myeloid cells in the spleens of naïve mice treated with PBS or 4 mg/Kg SW for 7 days. Ly6C and L6G cells are also $CD11b^{+}$. * indicates significant difference between experimental groups.
